# Formation of the toxic furan metabolite 2-butene-1,4-dial through hemin-induced degradation of 2,4-alkadienals in fried foods

**DOI:** 10.1186/s41021-025-00330-2

**Published:** 2025-04-08

**Authors:** Hiroshi Kasai, Kazuaki Kawai, Koichi Fujisawa

**Affiliations:** Department of Environmental Oncology, Institute of Industrial Ecological Sciences, University of Occupational and Environmental Health, 1-1 Iseigaoka, Yahatanishi-Ku, Kitakyushu, Fukuoka 807-8555 Japan

**Keywords:** 2,4-heptadienal, 2,4-decadienal, Furan, 2-butene-1,4-dial, Cysteine-pyrrole-lysine, Protein crosslink, Fried foods

## Abstract

**Background:**

The mechanism of protein modification by 2,4-alkadienals (ADE), lipid peroxidation products prevalent in fried foods, was investigated through model reactions.

**Results:**

A mixture of 2,4-heptadienal (HDE) and hemin was initially incubated at pH 3.0–7.4, followed by treatment with acetyl-cysteine (AcCys) and acetyl-lysine (AcLys) at pH 7.4. Analysis via HPLC revealed a product with a characteristic UV spectrum as the primary peak. This product was identified as an AcCys-pyrrole-AcLys (CPL) crosslink derived from AcCys, 2-butene-1,4-dial (BDA), and AcLys. Increasing the HDE concentration in the initial reaction led to maximum CPL formation at pH 3.5 in the presence of hemin. Lowering the HDE concentration with a higher Cys/HDE ratio resulted in CPL formation, which was observed at pH 7.4 and 3.5 in the presence of hemin. Upon incubation of ADE and hemin at pH 3.0–3.5, BDA was directly identified as 2,4-dinitrophenylhydrazone. BDA was also detected in the 2,4-decadienal reaction mixture. Additionally, a notable propensity for high BDA-dC adduct formation with hemin under acidic conditions was observed, consistent with the results of CPL assay and BDA–2,4-dinitrophenylhydrazone analysis.

**Conclusions:**

1) BDA is efficiently generated from ADE in the presence of hemin under gastric conditions, and 2) BDA-derived CPL can also form under physiological conditions (pH 7.4) through the interaction of ADE, hemin, Cys, and Lys. BDA is recognized as the primary reactive metabolite of the suspected carcinogen furan (IARC, 2B). Given that human intake of ADE exceeds that of furan and acrylamide (IARC 2A) by several orders of magnitude, and the estimated hemin concentration in the stomach post-meal is comparable to the present study, a substantial amount of BDA may form in the stomach following consumption of fried foods and meat. The risk assessment of ADE warrants a thorough re-evaluation, based on the toxicity mechanism of BDA.

**Supplementary Information:**

The online version contains supplementary material available at 10.1186/s41021-025-00330-2.

## Introduction

High consumption of deep-fried foods is associated with various adverse health effects, such as cancer, atherosclerosis, and Alzheimer’s disease [1]. 2,4-Alkadienals (ADE) are significant toxic byproducts of lipid peroxidation commonly found in fried foods [2]. Heat-induced oxidative degradation of linolenoylglycerol and linoleoylglycerol leads to the production of 2,4-heptadienal (HDE) and 2,4-decadienal (DDE), respectively. Our previous findings indicated that the hemin-induced oxidation of ADE forms 6-hydroxy- and 6-oxo-alkadienals, which are implicated in the generation of mutagenic DNA legion 1,2-ethenoguanine [3]. Expanding on our previous research, we investigated protein modifications, which play a significant role in the pathogenesis of such chronic diseases [4]. It has been reported that the interaction of DDE with proteins leads to modifications in Lys and Cys residues [5]. In this study, we examined the reaction of ADE, acetyl-cysteine (AcCys), and acetyl-lysine (AcLys) in the presence of hemin as a model for protein modification. The reasons for the use of hemin in the reaction are as follows: 1) the common consumption of meat and fried foods, 2) the release of hemin from heme-proteins during gastric digestion [6], and 3) the catalytic role of hemin in lipid peroxidation [3, 4].

### Experimental

#### Materials

HDE (90%), DDE (90%), t-butylhydroperoxide (tBuOOH) (70%), and fumaraldehyde bis(dimethyl)acetal were purchased from Tokyo Chemical Industry Co., Ltd. (Tokyo, Japan). 2,4-Dinitrophenylhydrazine (DNPH) is a product of FUJIFILM Wako Pure Chemical Corporation (Osaka, Japan). Hemin, N-AcCys, N-AcLys, and 2’-deoxycytidine (dC) were acquired from Sigma-Aldrich Chemical Co., USA.

### Model reactions of HDE, hemin, AcCys, and AcLys

#### Method A

The hemin was dissolved in 20 mM NaOH (2.17 mg/mL). HDE (16.7 µL, 120 µmol) was dissolved in 1.2 mL of acetonitrile. A mixture of the HDE solution (33 µL, 3.3 µmol), acetonitrile (67 µL), hemin (5.5 µL, final concentration = 60 µM), 2 M NaOAc (pH 4.5) (11.3 µL, final concentration = 75 mM), and water (183 µL) in a capped Eppendorf tube was incubated at 37°C for 4 h (mixture A). The reaction mixture (40 µL), 100 mM phosphate buffer (pH 7.4) (100 µL), solutions of AcCys (20 mM), and AcLys (20 mM) (20 µL, each) were mixed and incubated at 37°C for 39 h (second reaction). A 15 µL portion of the mixture was injected into the HPLC apparatus (Hewlett-Packard 1100 system connected with a photodiode array UV detector) with a column (Shiseido Capcell Pak C-18, 4.6 × 100 mm, 5 µm) under the following conditions: temperature, 40°C; flow rate, 0.5 mL/min; elution, linear gradient of ethanol concentration in 5 mM ammonium formate. The gradient was as follows: 0–20 min, 0%–23%; 20–40 min, 23%–77%.

#### Method B

A mixture (303 µL) containing HDE (4.2 µL, 30 µmol) and other components was incubated for the first reaction as specified for Method A. The second reaction was conducted using Method A for 18 h. A 20 µL aliquot of each mixture was injected into the HPLC column. Elution involved a linear gradient of ethanol concentration in 5 mM ammonium formate over 10–60 min, ranging from 0 to 77%.

#### Method C

The reaction was conducted as specified for Method B, except that a mixture (300 µL) containing a diluted solution of HDE (16.5 µL, 1.7 µmol) and other components was incubated for the first reaction.

#### Method D

HDE (16.7 µL) was dissolved in 1.2 mL of 25% aqueous acetonitrile. A 1 mL aliquot of the solution was injected into the HPLC column. Experimental conditions were as follows: column: Shiseido Capcell Pak C18, 10 × 250 mm, 5 µm; elution: linear-gradient of ethanol, 0–60 min, 15%–50%; speed, 2 mL/min. The HDE fractions eluted between 51 and 53 min were collected. The reaction was conducted as specified for Method B, except that a mixture (292 µL) containing the purified HDE fraction (100 µL, 1.7 µmol) and other components was incubated for the first reaction.

Reaction conditions for the conversion of HDE into CPL (Methods A, B, C, and D) are summarized in the Table 1.

**Table 1:** Reaction conditions for the conversion of HDE into CPL

Method	First reaction			Second reaction			
	Concentration of HDE	Concentration of hemin	pH	Concentration of HDE^*^	Concentrations of AcCys, AcLys	Reaction time	pH
A	10.9 mM	60 µM	pH 4.5	2.40 mM	2.2 mMAcCys 2.2 mMAcLys	39 h	pH 7.4
B	99 mM (High HDE)	59.4 µM	pH 3.5 pH 7.4	21.8 mM	2.2 mMAcCys 2.2 mMAcLys	18 h	pH 7.4
C	5.67 mM (Low HDE)	60 µM	pH 3.5 pH 7.4	1.25 mM			
D	5.85 mM (Low HDE Purified)	62 µM	pH 3.5 pH 7.4	1.28 mM			

^*^HDE concentrations of the second reaction mixture, calculated from initial HDE concentrations

### Preparation of standards for BDA, AcCys-pyrrole-AcLys, and BDA-dC adducts

To prepare fumaraldehyde (2-butene-1,4-dial, BDA), fumaraldehyde bis(dimethyl acetal) (5 µL) was added to a 1 mM HCl solution (995 µL) and incubated at 22°C for 30 min in the dark. To prepare AcCys-pyrrole-AcLys (CPL), the BDA solution obtained (25 µL) was then mixed with 20 mM solutions of AcCys (50 µL) and AcLys (50 µL) in 100 mM phosphate buffer (pH 7.4) and reacted at 37°C for 4 h, following the method described by Munko et al. [7]. The product was purified using HPLC, with the main product eluting at approximately 22 min under the same conditions as those of Method B.

The BDA-dC adduct was prepared using the method described previously [8]. Confirmation of the BDA-dC adduct formation was achieved through mass spectrometry (positive ion mode, 312.1188 and 312.1189 for the two isomers; MW = C_13_H_17_N_3_O_6_ = 311).

### Analysis of BDA–DNPH adducts generated in model reactions

DNPH (5.2 mg) was dissolved in 4.16 mL of acetonitrile and mixed with 4.2 µL of 6 N HCl. After storing the mixture overnight at 20–22°C, a small amount of precipitate was formed at the bottom. The supernatant was then utilized for the reaction. At various time points of the first reaction (Method B), a 10 µL portion of the mixture was added to the DNPH solution (90 µL) and reacted at 20–22°C for 40 min in the dark. A 10 µL aliquot of reaction mixture B was injected into the HPLC column to analyze the mono-DNPH–BDA adduct. Elution conditions included a linear gradient of ethanol concentration in water: 0–60 min, 38.5%–77%.

Under the same conditions, BDA standards of various concentrations were reacted with DNPH and analyzed using HPLC to establish a calibration curve. The BDA level in the pH 7.4 reaction mixture was further confirmed by reacting 10 µL of the sample with 100 µL of 0.2 M acetate buffer (pH 3.5) and 90 µL of DNPH solution at 20°C for 40 min.

In addition to Method B, for the reaction under pH 3.0 condition, 4 µL of 0.1 N HCl was added instead of the buffers to prepare 303 µL of solution, and DDE (5.8 µL, 30 µmol) was used instead of HDE. In certain reactions, 1 µL of tBuOOH (final concentration = 24 mM) was added as a model of lipid hydroperoxides commonly found in fried foods. The pH levels of all reaction mixtures were verified using a pH meter equipped with a microelectrode.

### Analysis of BDA-dC adducts formed in model reactions

The first reaction mixture of Method B or C (40 µL), 10 mM dC (90 µL), and 2 M phosphate buffer (10 µL) was combined and then incubated at 37°C for 17 h. A 20 µL sample of the reaction mixture was analyzed using HPLC. The elution parameters matched those outlined in Method B.

### Liquid chromatography-tandem mass spectrometry

The primary product, a’, and synthetic CPL were analyzed using liquid chromatography-tandem mass spectrometry (LC–MS/MS) with a UHPLC system (UltiMate 3000, Thermo Fisher Scientific, Yokohama, Japan) coupled to a hybrid quadrupole-Orbitrap mass spectrometer (Thermo Scientific Q Exactive Focus) and equipped with heated electrospray ionization (ESI, HESI-II). The sample separation was achieved on a L-column 3 C18 (2.1 mm × 100 mm, 3 µm, CERI, Tokyo, Japan) with a flow rate of 0.2 mL/min and a column temperature of 30°C. Mobile phase A contained 10 mM ammonium formate, while mobile phase B was composed of methanol. A linear gradient program over 40 min was used for separation, with the percentage of methanol in solvent B varying as follows: 0 min, 0%; 1–20 min, 0%–30%; 20–25 min, 30%–90%; 30–30.5 min, 90%–0%. The injection volume for the measurements was 5 µL. The ESI source was maintained at a temperature of 400°C, with sheath gas and auxiliary gas pressures set at 35 and 10 arbitrary units, respectively. The ion-spray voltage was 3.5 kV, the capillary temperature was 320°C, and the S-lens radio frequency level was 70. Data for the primary product a’ and synthetic CPL were acquired by polarity switching in the AIF mode at CEs of 10, 20, and 30. Data for BDA–DNPH were acquired by negative ions using the AIF mode at CEs of 10, 20, and 30.

## Results

### Identification of a reaction product of HDE, hemin, AcCys, and AcLys (Method A)

**Fig. 1:**
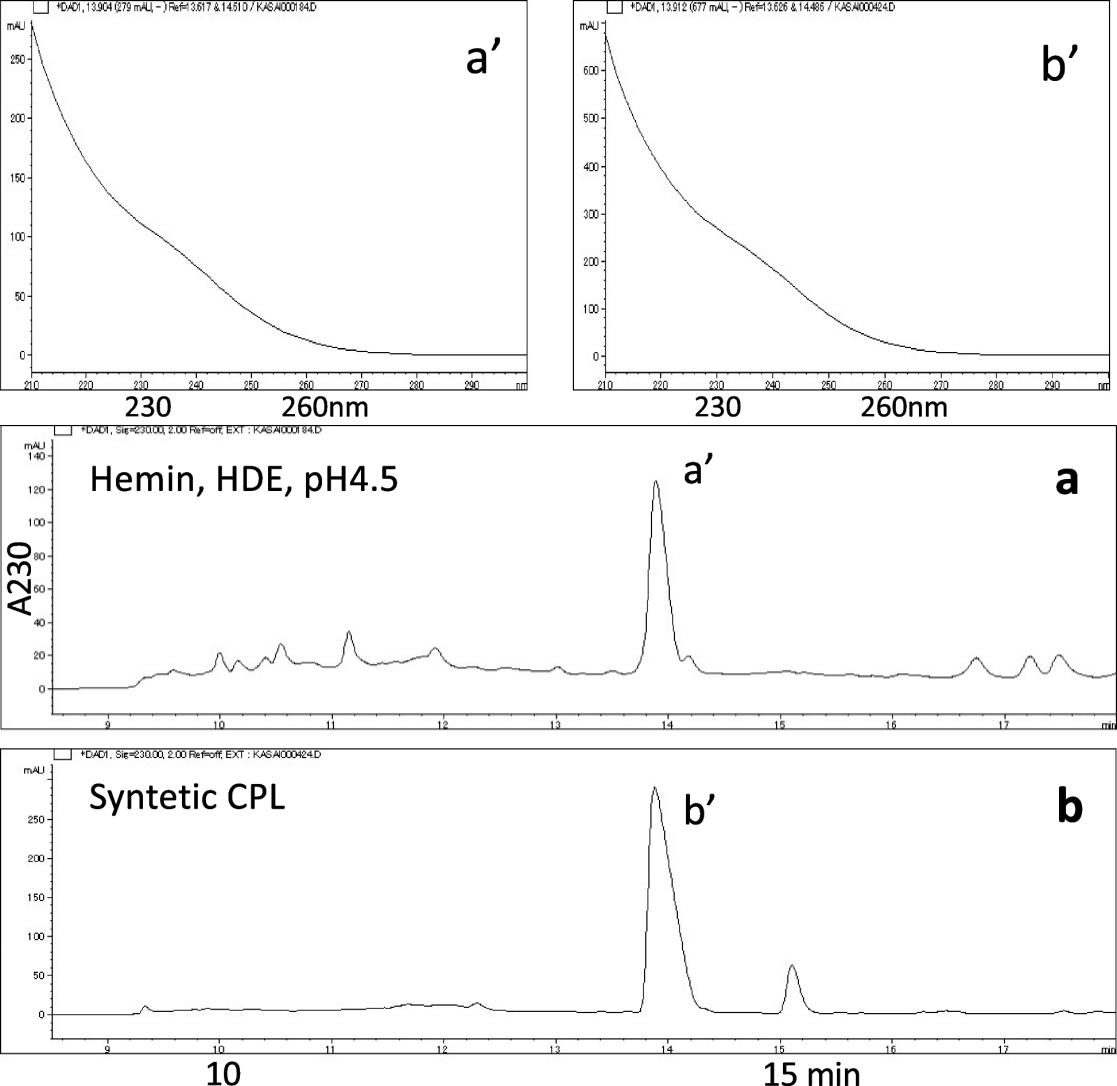
Isolation and identification of AcCys-pyrrole-AcLys (CPL) in the reaction mixture of (hemin, HDE, pH 4.5), followed by the addition of AcCys and AcLys. **a** Analyses of the reaction mixture and (**b**) synthetic CPL. The upper figures display the UV spectra of peaks a’ and b’

**Fig. 2:**
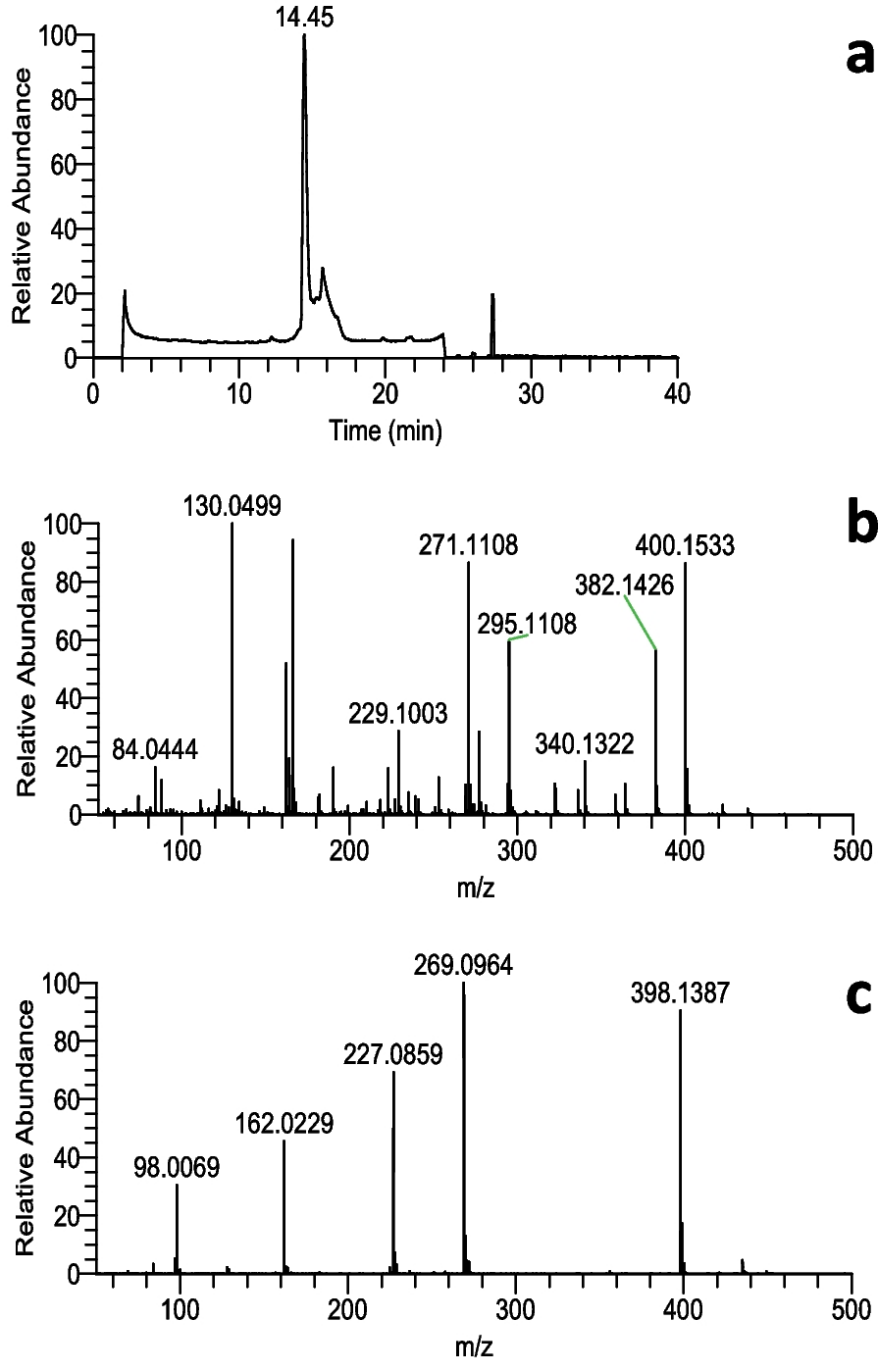
Mass spectra of the primary product a’ in (**a**) Mass chromatogram (negative TIC). **b** Mass spectrum (positive). **c** Mass spectrum (negative)

Model reactions of HDE were initially conducted at pH 4.5 with or without hemin, followed by reactions with AcCys and AcLys at pH 7.4. The results of HPLC analysis of the reaction mixture revealed one prominent product with a retention time of 14 min, displaying a distinct UV spectrum characterized by strong end-absorption in a short-wavelength region (Fig. 1). This peak was not observed when either AcCys or AcLys was individually added to the reaction mixture, implying the necessity of both components for its formation. The mass spectrum of peak a’ in Fig. 1a (positive, m/z 400.1533; negative, m/z 398.1387; elemental composition, C_17_H_25_N_3_O_6_S = 399) indicates that AcCys (MW = 163), AcLys (MW = 188), and fumaraldehyde (2-buten-1,4-dial, BDA) (MW = 84) combined through the loss of two water molecules (163 + 188 + 84–36 = 399). The UV and mass spectra of peak a’ in Fig. 1a closely resemble the published data for AcCys-pyrrole-AcLys (CPL) [8, 9]. Peak a’ in Fig. 1a exhibited identical HPLC retention time and UV spectrum to peak b’ of the synthetic CPL (Fig. 1b). Furthermore, the mass spectra of peak a’ (Fig. 2) and the synthetic standard (Suppl-1) were also found to be identical.

### Model reactions using high concentrations of HDE (Method B)

**Fig. 3:**
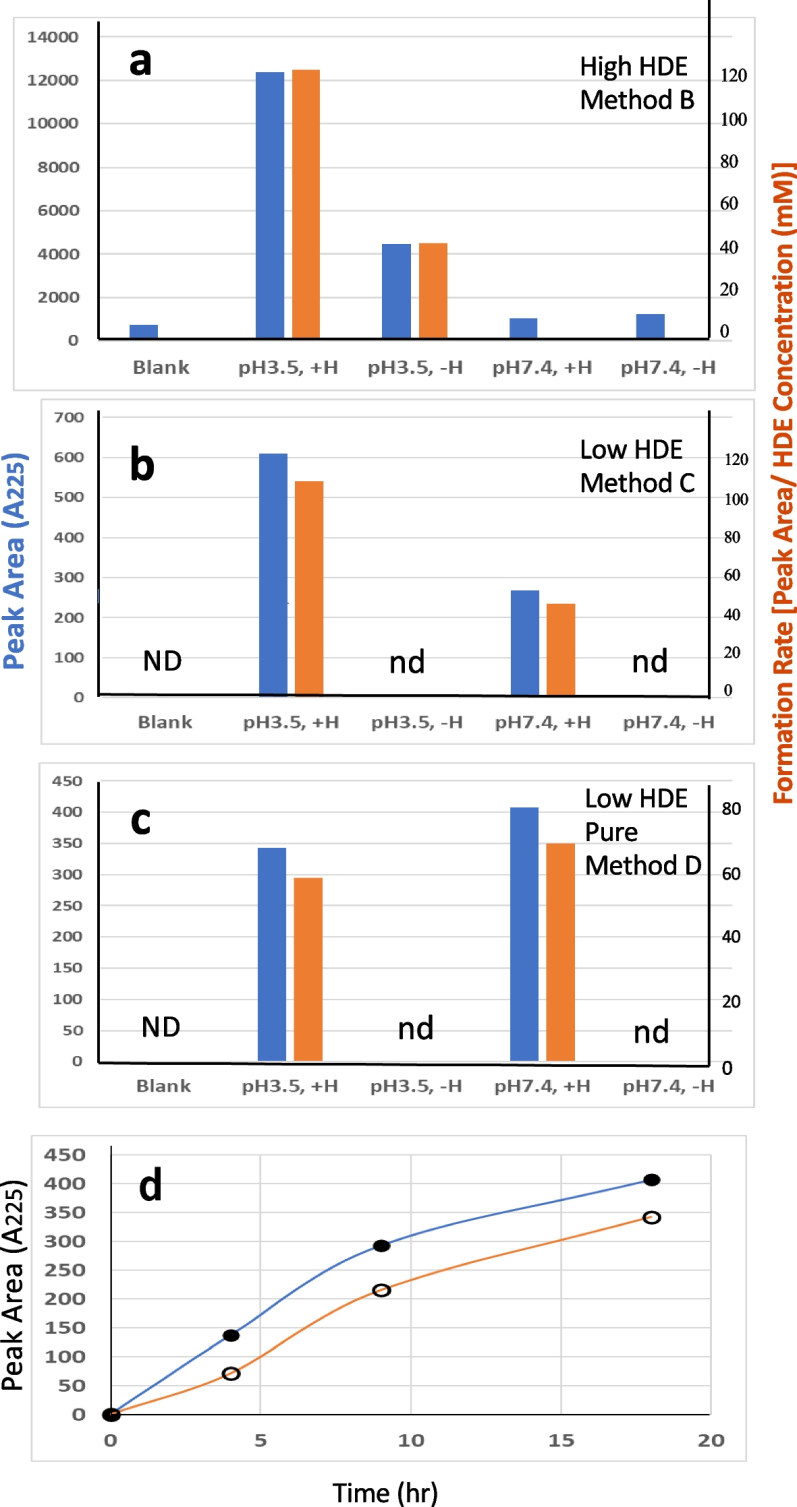
Summary of the formation of CPL using different concentrations of HDE: **a** high HDE concentration (Method B), **b** low HDE concentration (Method C), and (**c**) low HDE concentration without additives (Method D). **d** Time course of CPL formation using Method D, with data points for pH 7.4 (●), pH 3.5 (〇), no data (NO), and not detected (nd). The blue bar denotes the peak area. The orange bar indicates the formation rate [peak area / initial HDE concentration (mM)]

Using an excess amount of HDE, model reactions were conducted under various preincubation conditions, such as those representing a typical gastric environment (pH 3.5) or a cellular environment (pH 7.4), with or without hemin. Subsequently, the mixtures were incubated with AcCys and AcLys, and the CPL formation under both conditions was compared (Suppl-2). The presence of hemin and acidic pH emerged as crucial factors influencing CPL formation. The yields estimated by peak area were in the following order: pH 3.5, with hemin > pH 3.5, without hemin > > pH 7.4, with hemin ~ pH 7.4, without hemin (Fig. 3a, blue bars).

### Model reactions using low concentrations of HDE (Method C and D)

Model reactions using low concentrations of HDE were conducted at pH 3.5 or pH 7.4, with or without hemin, followed by the reaction with AcCys and AcLys at pH 7.4 (Method C). The HDE amount in reaction mixture C was 18 times lower than in the reactions using high concentrations of HDE (method B). This outcome significantly differed from those of the reactions using high concentrations of HDE, possibly due to the increased AcCys/HDE ratio. CPL formation occurred at both pH 3.5 and 7.4 in the presence of hemin but not in its absence (Suppl-3a, b; Fig. 3b).

When purified HDE was utilized (method D), CPL also formed in a time dependent manner at pH 3.5 and 7.4 in the presence of hemin (Suppl-4a, b; Fig. 3c, d). The formation rates under pH 3.5 and 7.4 conditions were different between Methods C and D, possibly due to the inclusion of the radical scavenger ethanol in reaction mixture D. CPL formation under different conditions is summarized in Fig. 3. It is noteworthy that CPL formation rates (peak area / HDE concentration) are comparable between high- and low-HDE concentration reactions (Fig. 3, orange bars). For example, the formation rates of the Method B (high HDE), C (low HDE), and D (low HDE, pure) reactions with the conditions of pH 3.5 and hemin were 125, 108, and 59, respectively.

### Analysis of BDA as 2,4-dinitrophenylhydrazone

**Fig. 4:**
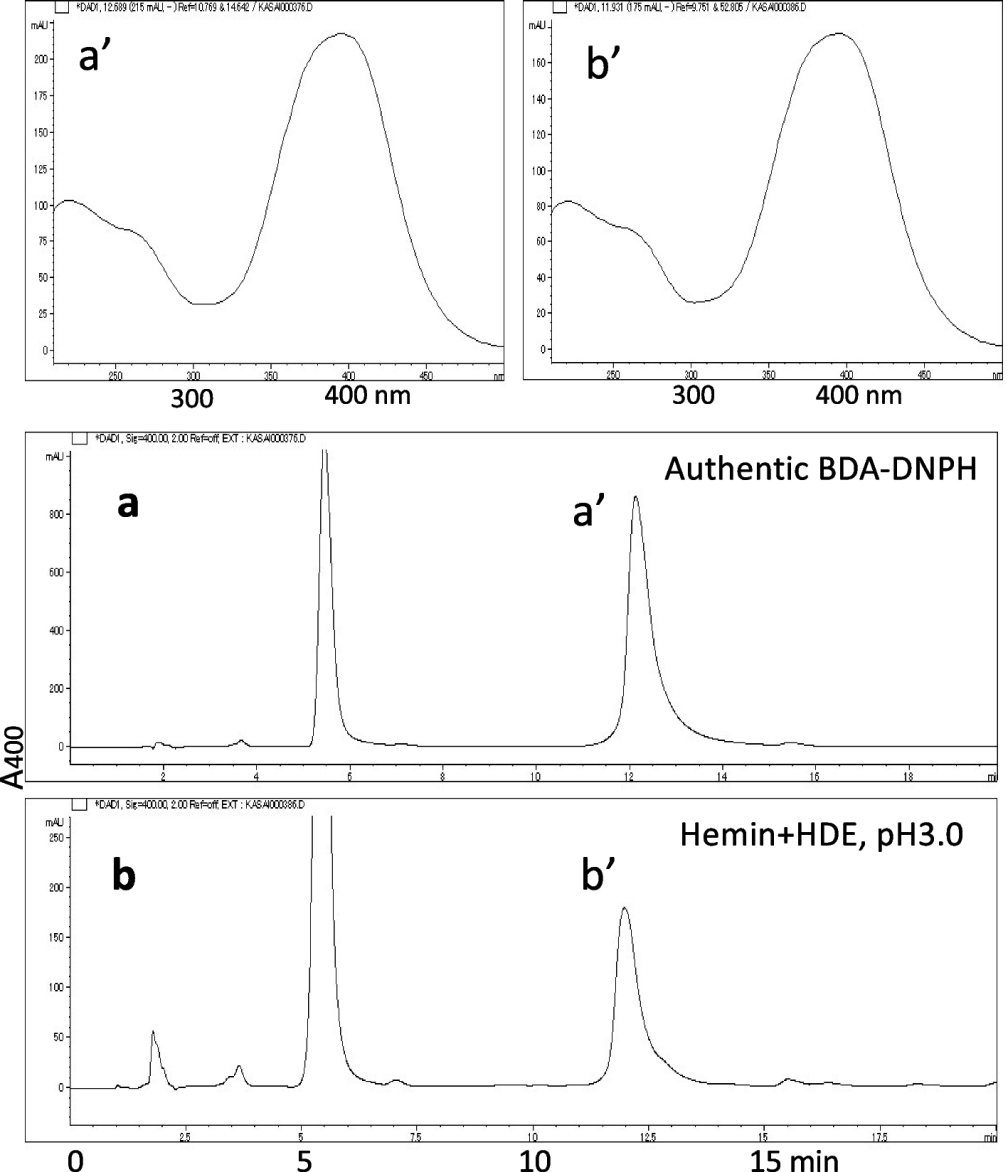
Detection of BDA as 2,4-dinitrophenylhydrazone: **a** analysis of authentic BDA–DNPH, **b** analysis of BDA formed in model reactions. The upper figures display UV spectra of peaks a’ and b’

**Fig. 5:**
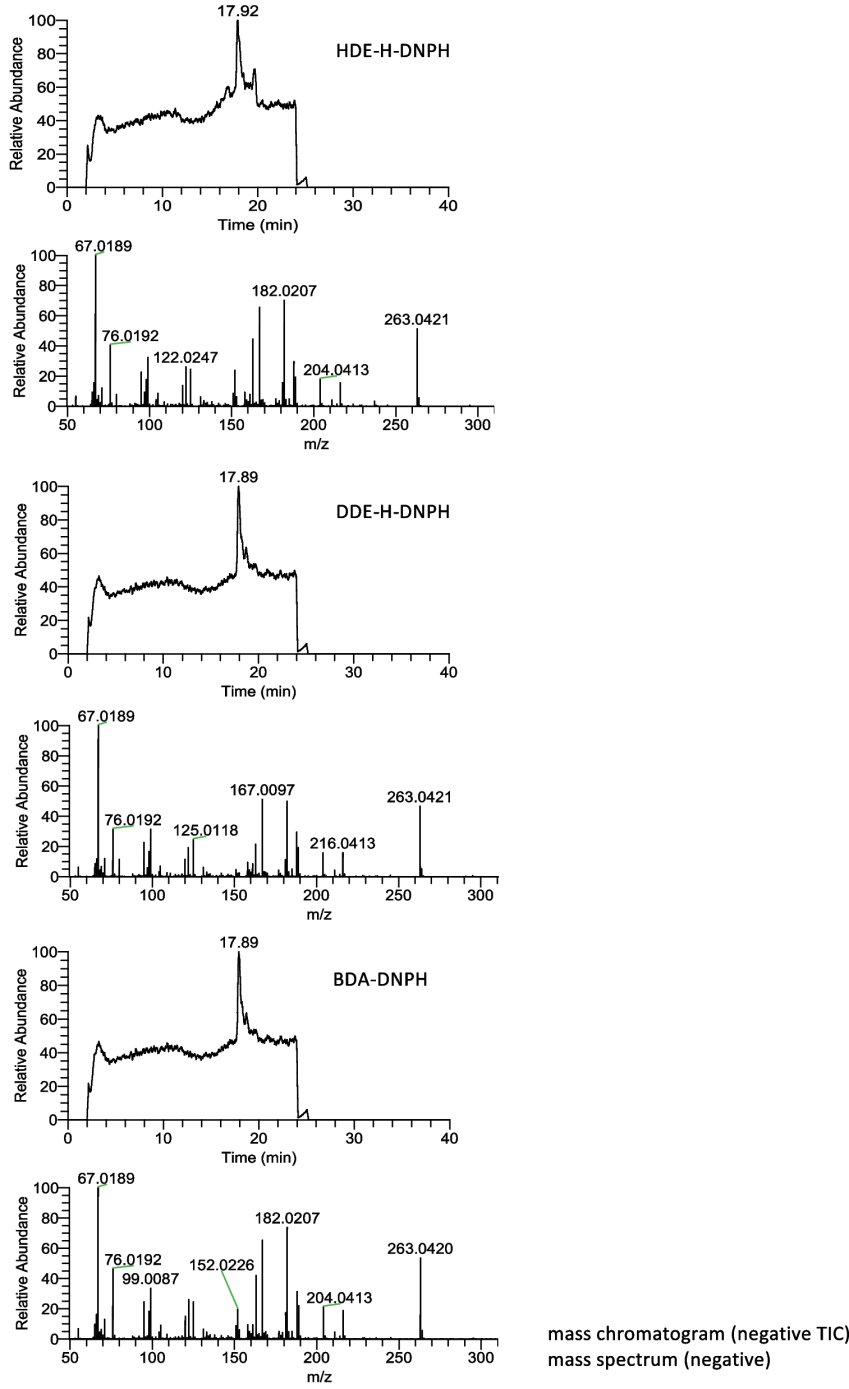
Mass spectra of BDA–DNPH obtained from reactions of HDE and DDE, and from standard BDA

**Fig. 6:**
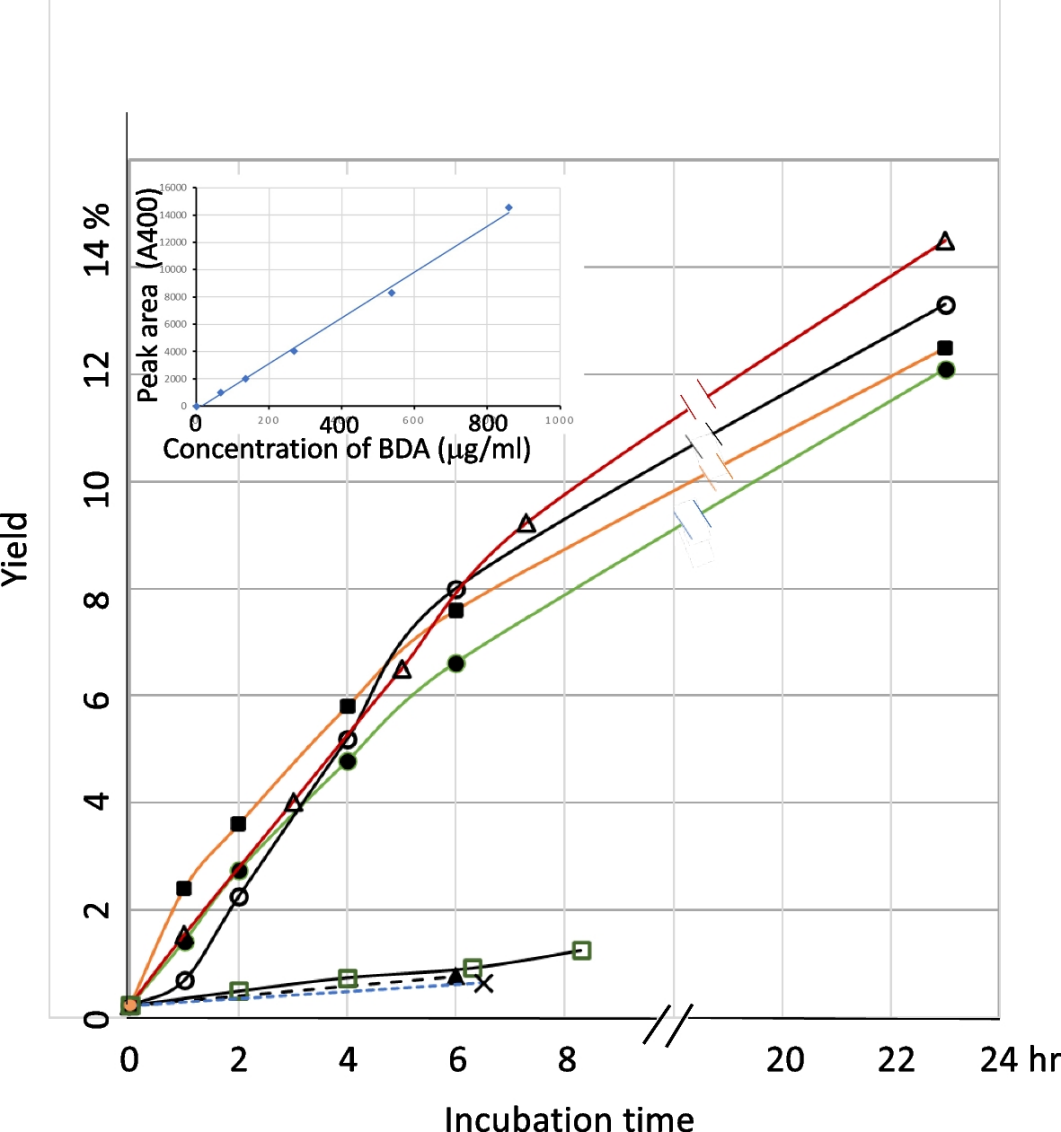
Formation of BDA from ADE under various conditions analyzed using the DNPH method. △, HDE, pH 3.5, + hemin; □, HDE, pH 3.5, -hemin; 〇, DDE, pH 3.0, + hemin; ●, HDE, pH 3.0, + hemin; ■, HDE, pH 3.0, + hemin, + tBuOOH; ▲, HDE, pH 7.4, + hemin; ×, HDE, pH 7.4, -hemin; Inset: calibration curve plotting BDA concentration on the horizontal axis and the peak area of BDA–DNPH on the vertical axis

Conversion of fumaraldehyde bis(dimethylacetal) to fumaraldehyde was examined using varying concentrations of HCl (0.12, 0.33, 1, 3, and 10 mM) at room temperature for 30 min. The conversion rate was the highest in the presence of 1 and 3 mM HCl. It was hypothesized that dimethyl acetal groups were hydrolyzed completely under this condition. The derivatization of BDA with DNPH was conducted at 20–22°C for 40 min, following the procedure described in the Experimental Section. Under these conditions, BDA was converted to BDA–mono-DNPH (Fig. 4), as confirmed by mass spectral analysis (Fig. 5). The calibration curve displayed excellent linearity (Fig. 6, inset). Stronger conditions, such as elevated DNPH concentrations and prolonged reaction times, resulted in the generation of red, insoluble, polymer-like precipitates.

With the simulated gastric digestion conditions (pH 3.0–3.5), we observed time-dependent BDA formation from HDE in the presence of hemin using the DNPH method (Fig. 6). The maximum yield was approximately 9% at 7 h (14% at 23 h). In the absence of hemin, the yield was minimal regardless of pH. BDA was also identified in a DDE reaction mixture at pH 3.0 in the presence of hemin. Upon adding tBuOOH to the reaction mixture at pH 3.0, a model of lipid hydroperoxides in fried foods [10], we observed a slight enhancement in the BDA yield compared to that in the absence of tBuOOH.

### Analysis of BDA-dC adducts

**Fig. 7:**
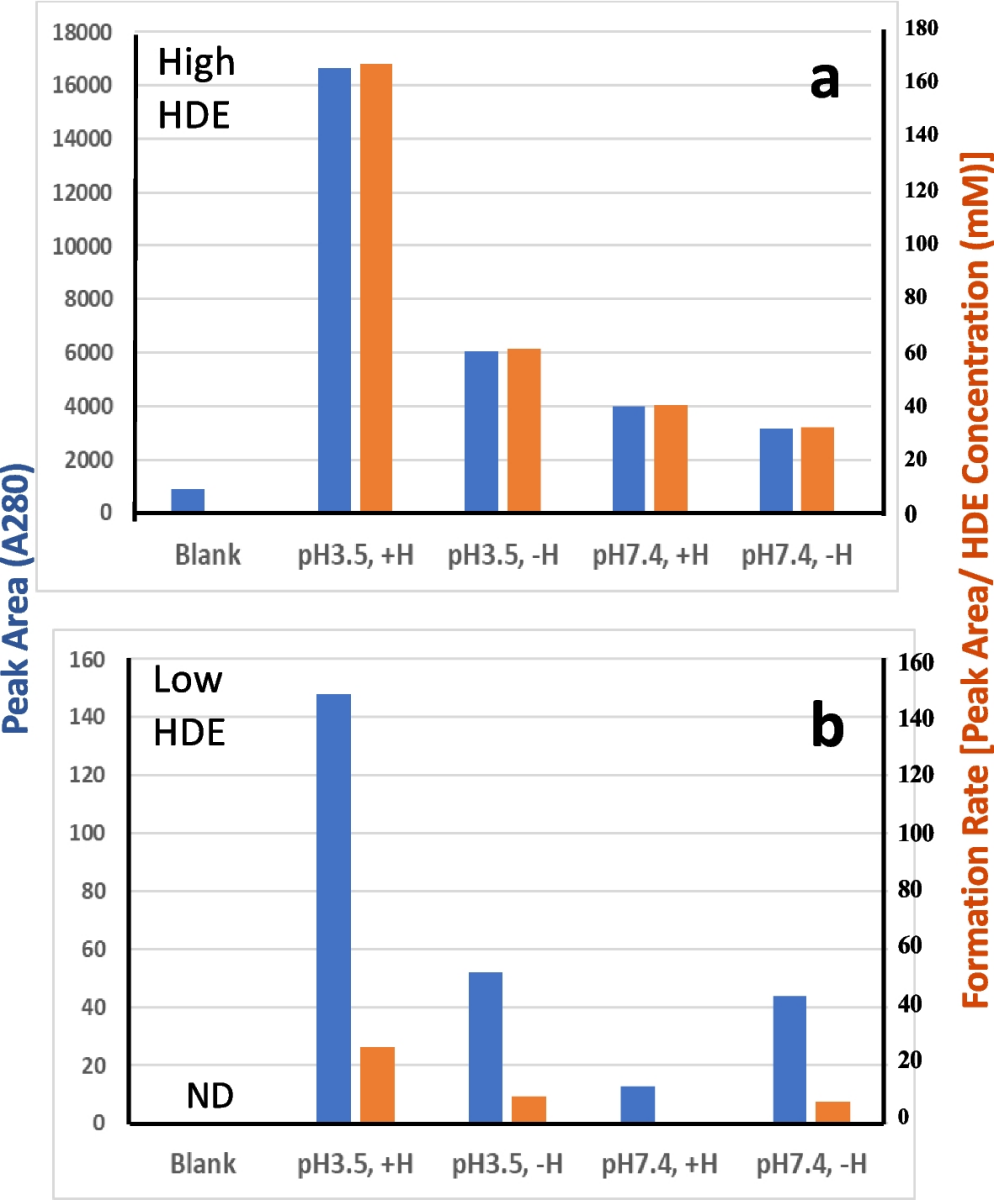
Summary of BDA-dC adduct formation: **a** reaction involving high concentrations of HDE (mixture B + dC); **b** reaction involving low concentrations of HDE (mixture C + dC); NO, no data. The blue bar denotes the peak area. The orange bar indicates the formation rate [peak area / initial HDE concentration (mM)]

As the CPL formation profile varied under different reaction conditions, such as pH levels and HDE concentrations, the BDA-dC adduct formation was investigated using the initial reaction mixtures B and C. The formation of the BDA-dC adduct was confirmed by comparing UV spectra and retention times in HPLC with those of a reference sample (Suppl-5, 6). Regardless of the HDE concentration, the highest peak area of the BDA-dC adduct was observed (Suppl-5, 6, Fig. 7, blue bars) at pH 3.5 in the presence of hemin, which is consistent with the results of the CPL and DNPH assays with high concentrations of HDE. However, the formation rates of BDA-dC (area / HDE concentration) in low HDE concentration reactions (Fig. 7b, orange bars) were significantly lower than those of high HDE concentration reactions (Fig. 7a, orange bars). Adduct formation was minimal at pH 7.4 with hemin in the reaction using low concentrations of HDE (Suppl-6c, Fig. 7b). Therefore, CPL formation through BDA at low concentrations of HDE (pH 7.4, with hemin) seemed to be specific to the hemin-thiol system (Fig. 3b, c).

## Discussion

**Fig. 8:**
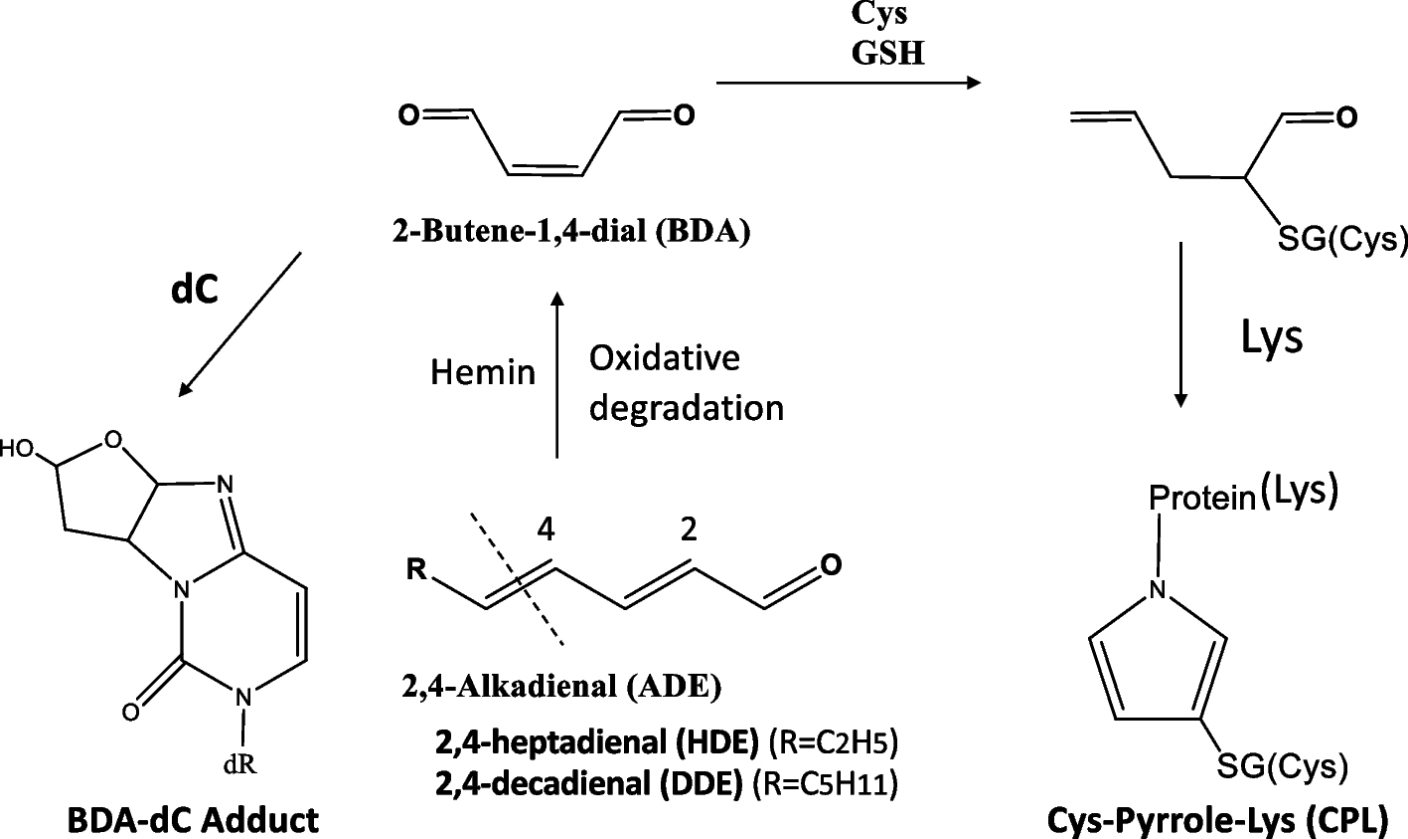
Formation of CPL and dC adducts from ADE via BDA

The transformation of HDE and DDE into BDA under oxygen-saturated conditions was documented by Lillard et al. [11] and Matthews et al. [12]. Zamora et al. investigated the degradation of HDE and DDE across a pH range of 2–11 at 200°C under air and detected BDA through GCMS [13]. Spiteller et al. detected small amounts of BDA through gas chromatography in autoxidized DDE within a phosphate buffer (pH 7.4) in the absence of iron ions, lacking quantitative analysis [14]. It is noteworthy that the toxicological mechanism of ADE via BDA formation has not been thoroughly explored despite early reports on ADE-to-BDA conversion. Based on the DNPH assay, substantial conversion of ADE to BDA, reaching a maximum of approximately 14%, was observed under gastric conditions (pH 3.0–3.5) in the presence of biologically relevant concentrations of hemin. The acid- and hemin-induced formation of CPL and dC adducts originating from BDA (Fig. 8) aligned with the results of the DNPH assay (Fig. 6).

**Fig. 9:**
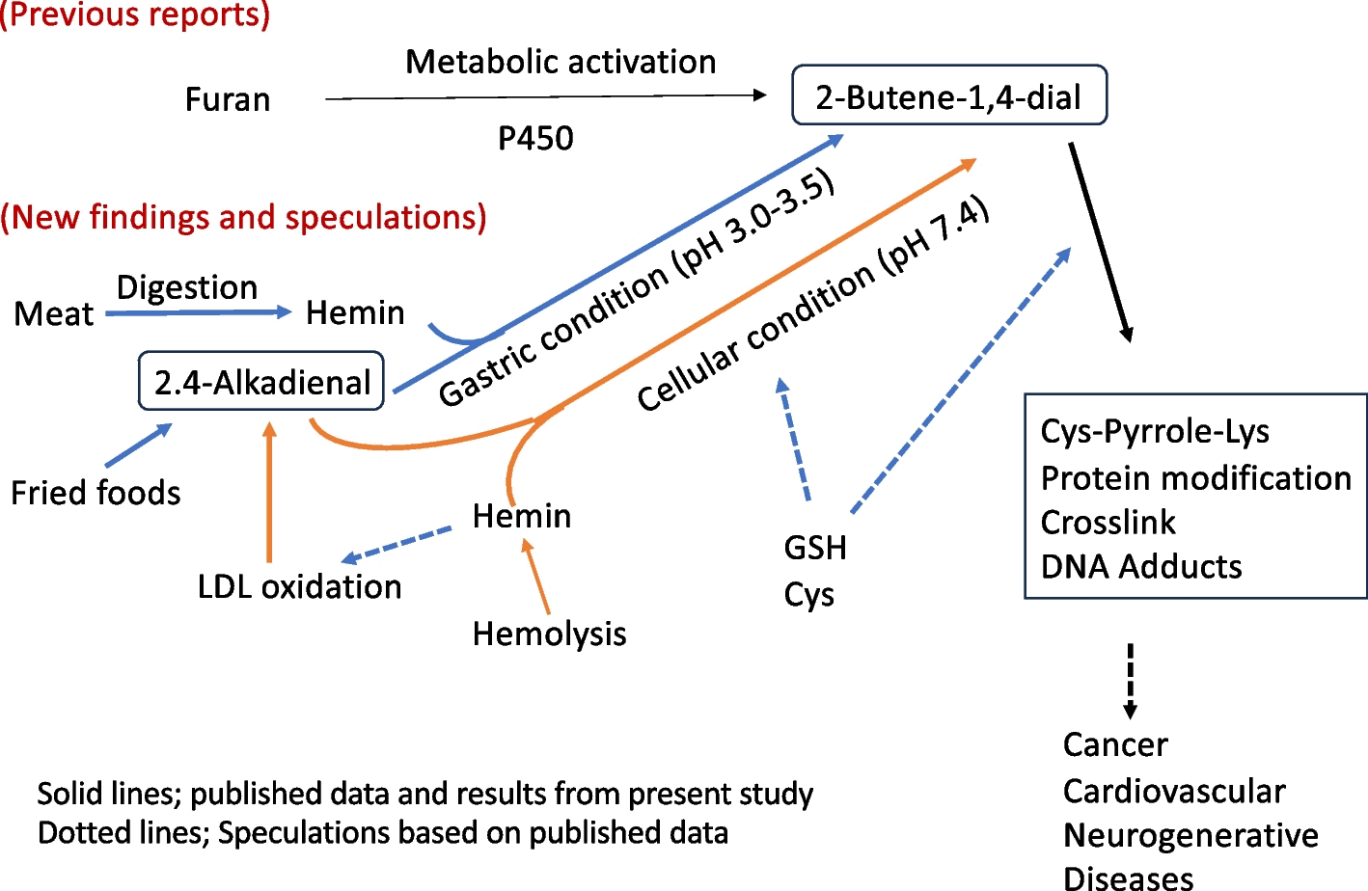
Summary and speculations

Toxic BDA is formed by P450-induced metabolic activation of furan (Fig. 9), which is classified as a possible human carcinogen Group 2B by the IARC [15, 16]. BDA reacts with DNA bases, particularly cytosines, to form adducts, demonstrating mutagenic activity [15]. BDA has been demonstrated to react with cellular nucleophiles such as GSH and amino acids and to cause cross-links between thiols and amino groups. Owing to the bifunctionality of BDA, intermediates resulting from the conjugation of BDA with GSH (or Cys) remain chemically reactive and can alkylate protein bound Lys to generate a Cys (GSH)-pyrrole-Lys (CPL) crosslink (Fig. 8) [15]. A CPL structure (GSH-BDA-Lys) has been identified in the histone tail of a furan-administered mouse liver, suggesting a potential role for epigenetic mechanism in furan carcinogenesis [17].

The results of the present study demonstrate that furan and ADE are transformed into a common reactive form, BDA, through metabolic activation in the former or through chemical transformation under stomach conditions in the latter (Fig. 9). Therefore, ADE likely possesses a carcinogenic potential comparable to furan.

Although ADEs are abundant lipid peroxidation products found in fried foods [2] and have mutagenic [18] and carcinogenic effects [19], their toxicity has been underestimated. The European Food Safety Authority concluded that genotoxicity can be ruled out in representative ADE derivatives [20]. For one of the lipid peroxide products, acrolein (IARC 2A), the World Health Organization (WHO) and the Australian Government Department of Health (AGDH) recommended maximal human daily intake (MHDI) values of 525 and 35 µg, respectively [21]; however, no information on the regulation of ADE is available. Surprisingly, DDE formation is recommended [22, 23], or it is added to foods [24] because of its flavor. Based on human intake of furan (0.34–1.23 µg/day/kg of body weight (BW)) [15] and ADE (83 µg/day/kg BW), calculated from a tentative daily consumption of fried foods (138 g) [21] and concentration of ADE (HDE + DDE) in French fries (42 mg/kg) [2], the consumption ratio of ADE/furan for 70 kg BW is 34–126 (molar base). Comparing the concentrations of ADE and the well-known suspected carcinogen acrylamide (Acr) (IARC 2A) [25] in French fries, the ratio of ADE/Acr was calculated to be 38 (molar base). Therefore, it is evident that the human intake of ADE is significantly higher than that of furan and Acr. Additionally, as the estimated concentration of hemin in the stomach after a meat-containing meal (60–160 µM) is comparable to that of the present study (60–62 µM) [26], a considerable amount of BDA may be formed from ADE in the stomach after fried food and meat meals. However, after the intake of a meat diet, high concentrations of hemin derivatives such as iron-ligated deuteroporphyrin and pemptoporphyrin have been detected in human feces [27]. Previous studies have also suggested that high dietary intake of heme iron increases the colonic levels of lipid-derived electrophiles, including 4-hydroxynonenal, malondialdehyde, and DDE, leading to an increased risk of colorectal cancer [28]. Therefore, BDA may form in the colon. BDA formed in the stomach and colon may directly interact with gastric and colonic tissues. ADE and BDA may also be absorbed from the gut into systemic circulation in vivo as other chemically-reactive α, β-unsaturated aldehydes [21] and may reach various organs. In fact, elevated levels of ADEs were detected in the plasma of the smoking/drinking group compared to the control group [29].

Epidemiological studies have indicated that increased consumption of deep-fried foods is associated with higher rates of gastric [30], colorectal [31], and prostate cancers [32]. In the context of this study, overconsumption of deep-fried meat has been linked to elevated incidences of breast [33], pancreatic [34], and lung [35] cancers. Consuming fried foods such as French fries and falafel poses a risk for non-alcoholic fatty liver disease (NAFLD), the predominant type of chronic liver ailment and presently the second most common cause of hepatocellular carcinoma [36].

In addition to cancer, excessive consumption of fried foods can lead to cardiovascular and neurodegenerative diseases. For example, hemin, released due to hemolysis, is known to promote LDL oxidation and induce crosslinking and aggregation of ApoB protein, initiating atherogenesis [4]. HDE has also been identified in auto-oxidized LDL in laboratory settings [37]. Lipid peroxidation is closely linked to neurodegenerative conditions [38]. For example, hemin-mediated lipid peroxidation and protein crosslinking in myelin, a complex of proteins and lipids, have been implicated in the pathogenesis of Alzheimer's disease [39] and multiple sclerosis [40, 41]. Thus, protein modification and crosslinking by BDA, generated by ADE and hemin, may play a role in the pathogenesis of these diseases (Fig. 9).

In the present study, under cellular conditions at pH 7.4, the sequential reactions of 1) ADE + hemin (pH 7.4) and 2) + Cys + Lys (pH 7.4) produced CPL crosslink (Suppl-3b, Suppl-4b; Fig. 3b, c). The presence of hemin and thiol in the second reaction mixture may enhance reactive oxygen species (ROS) generation through a continuous Fenton-like process involving hemin/Fe3 +→hemin/Fe2 + recycling [42] and stimulate ADE→BDA degradation even at pH 7.4. Consequently, BDA might be created within the hemin-ADE-SH-O2 complex [43].

Global consumption of fried foods is high. Specifically, under gastric conditions, the primary lipid peroxidation products, ADEs, in fried foods can transform into BDA—a well-known toxic furan metabolite—in the presence of hemin. The result of the present study raises an increasing concern about the excess intake of fried foods. To mitigate ADE risk, potential strategies include 1) utilizing flying oils such as olive, palm, or canola oil as they produce lower ADE levels compared to polyunsaturated fatty acid rich oils such as sunflower, corn, or soyabean oil [2, 44], and 2) avoiding the repeated use of frying oil, which increases ADE levels [44]. Numerous epidemiological studies have indicated that a diet rich in vegetables and fruits offers protection against these cancers. Plant phenolics may capture ADE and BDA through carbonyl-phenol adduct formation [45, 46]. Urgent reevaluation of ADE risk is necessary based on the BDA formation mechanism. Further investigations are essential to validate the toxicity mechanisms in both animals and humans. Analyzing CPL-related biomarkers could prove beneficial in these studies. Additionally, it is crucial to continue researching cooking techniques to minimize ADE formation or explore food combinations that can deactivate ADE and BDA to prevent diseases induced by fried foods.

## Supplementary Information 

Additional file 1: Suppl-1. Mass spectra of synthetic CPL. a) Mass chromatogram (negative TIC). b) Mass spectrum (positive). Mass chromatogram (negative TIC). Suppl-2. Formation of CPL from high concentrations of HDE. The upper figures display the UV spectra of peaks a’, b’, and c’. Suppl-3. Formation of CPL from low concentrations of HDE (Suppl.). Inset: UV spectrum of the major peak. Suppl-4. Formation of CPL from low concentrations of HDE (purified). The upper figures display the UV spectra of peaks a', b', and e'. Suppl-5. Formation of BDA-dC adducts from high concentrations of HDE. Suppl-6. Formation of BDA-dC adducts from low concentrations of HDE.

## Data Availability

No datasets were generated or analysed during the current study.

## Abbreviations

ADE: 2,4-Alkadienal
HDE: 2,4-Heptadienal
DDE: 2,4-Decadienal
AcCys: Acetyl cysteine
AcLys: Acetyl lysine
BDA: 2-Butene-1,4-dial
CPL: AcCys-pyrrole-AcLys
dC: 2’-Deoxycytidine
DNPH: 2,4-Dinitrophenylhydrazine
tBuOOH: t-butylhydroperoxide
GSH: Glutathione
BW: Body weight
IARC: International Agency for Research on Cancer
WHO: Word Health Organization
