# Supplementary material for: Formation of the toxic furan metabolite 2-butene-1,4-dial through hemin-induced degradation of 2,4-alkadienals in fried foods
Source: Genes Environ. 2025 Apr 8;47:8. doi: 10.1186/s41021-025-00330-2 (PMC11978195; doi:10.1186/s41021-025-00330-2)
Supplement: Supplementary file 1 — Additional file 1: Suppl-1. Mass spectra of synthetic CPL. a) Mass chromatogram (negative TIC). b) Mass spectrum (positive). Mass chromatogram (negative TIC). Suppl-2. Formation of CPL from high concentrations of HDE. The upper figures display the UV spectra of peaks a’, b’, and c’. Suppl-3. Formation of CPL from low concentrations of HDE (Suppl.). Inset: UV spectrum of the major peak. Suppl-4. Formation of CPL from low concentrations of HDE (purified). The upper figures display the UV spectra of peaks a', b', and e'. Suppl-5. Formation of BDA-dC adducts from high concentrations of HDE. Suppl-6. Formation of BDA-dC adducts from low concentrations of HDE. [file 41021_2025_330_MOESM1_ESM.pdf]

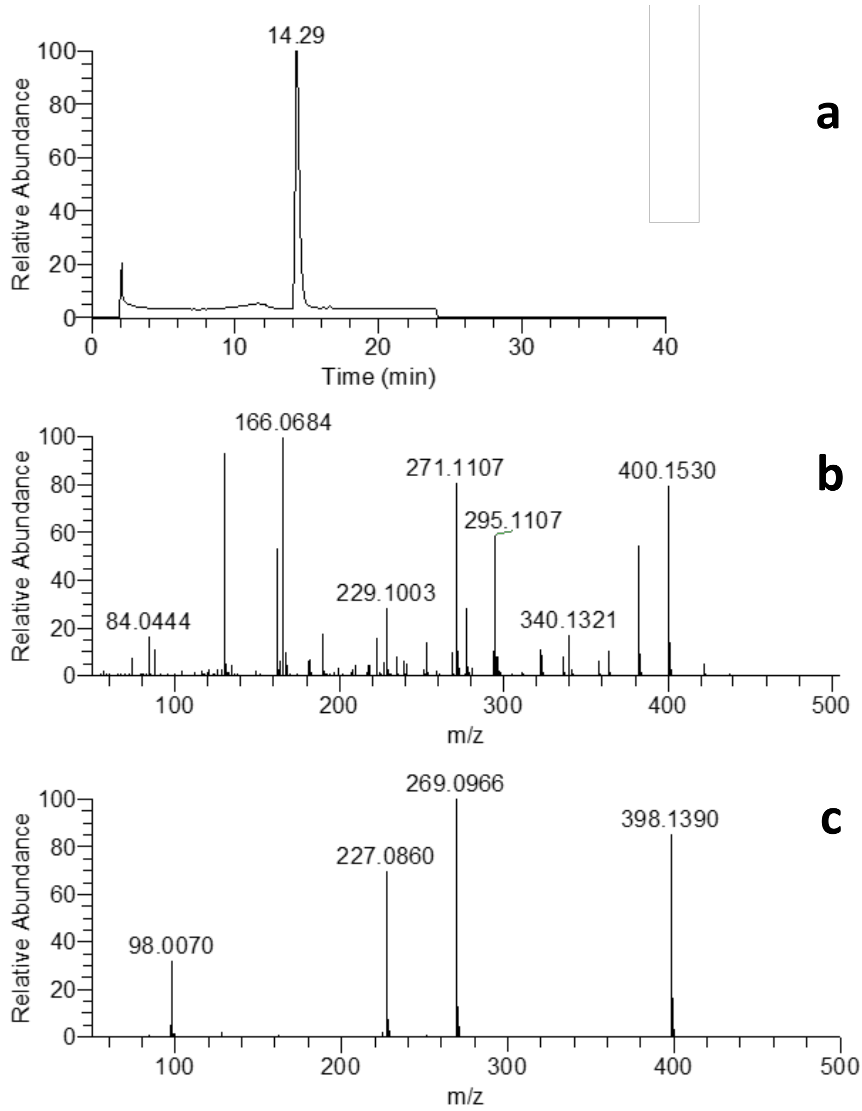

Suppl-1, Mass spectra of synthetic AcCys-Pyrrole-AcLys

a: Mass chromatogram (negative TIC); b: Mass spectrum (positive); c: Mass chromatogram (negative TIC)

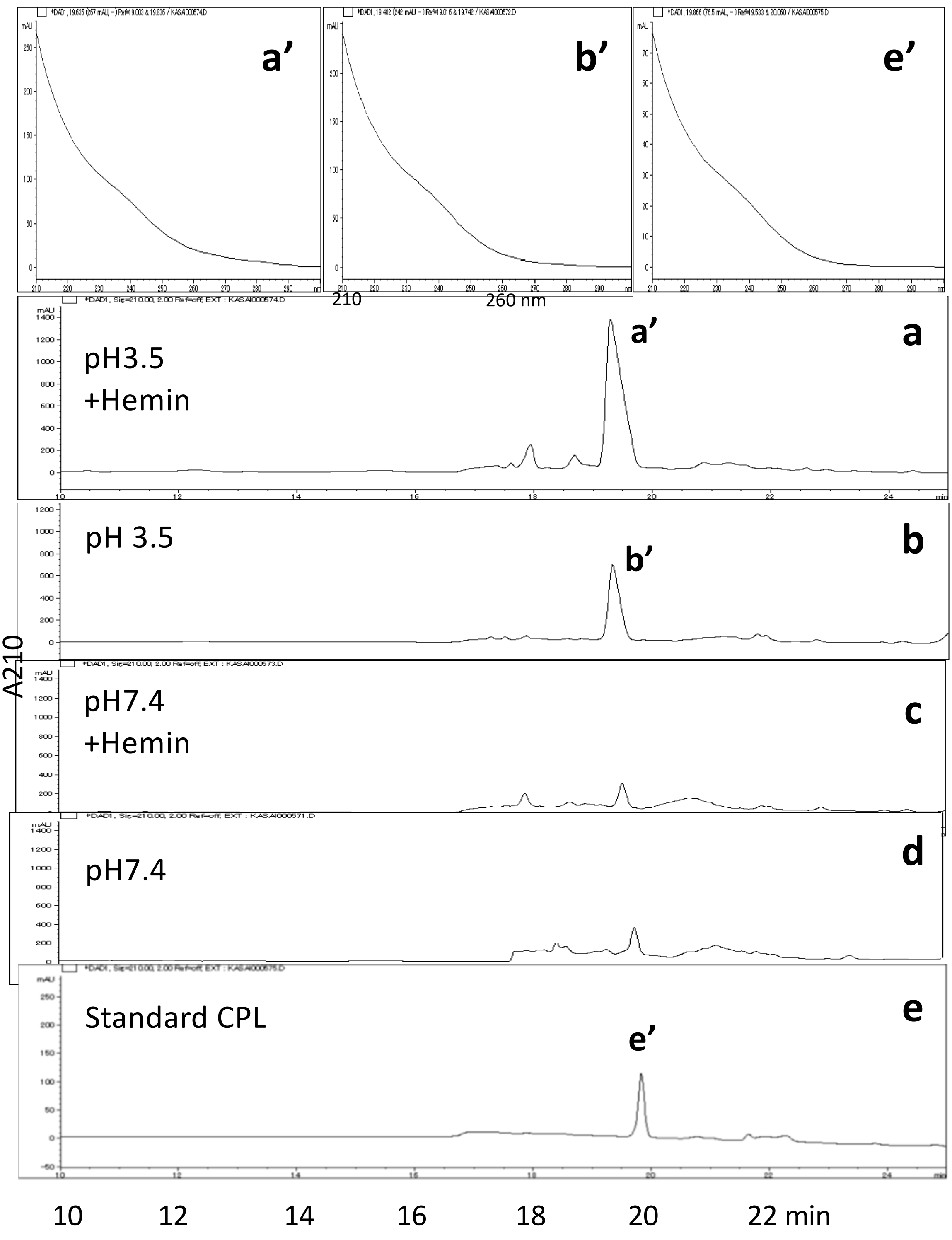

Suppl-2, Formation of CPL from high concentrations of HDE

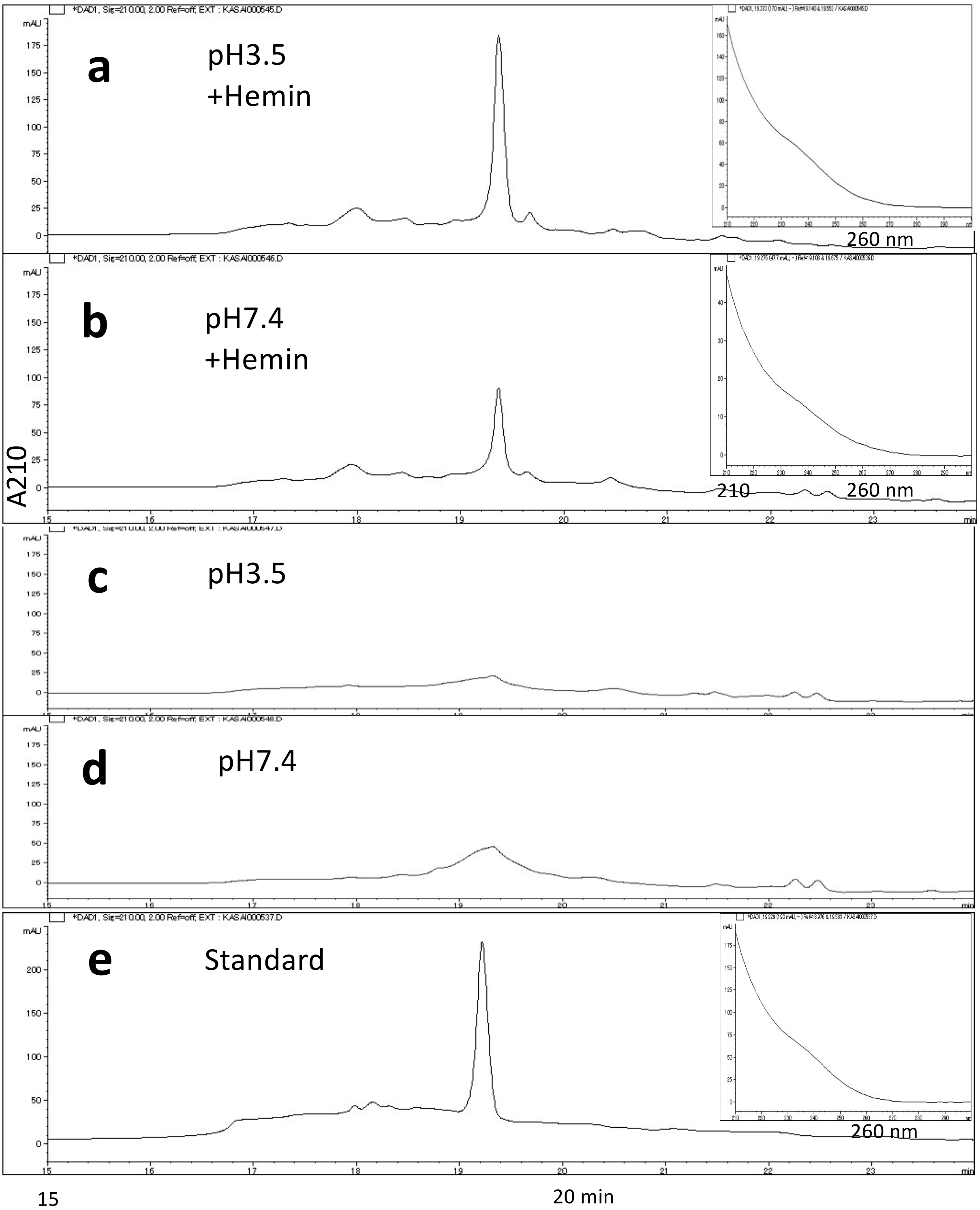

Suppl-3, Formation of CPL from low concentrations of HDE (Suppl.)

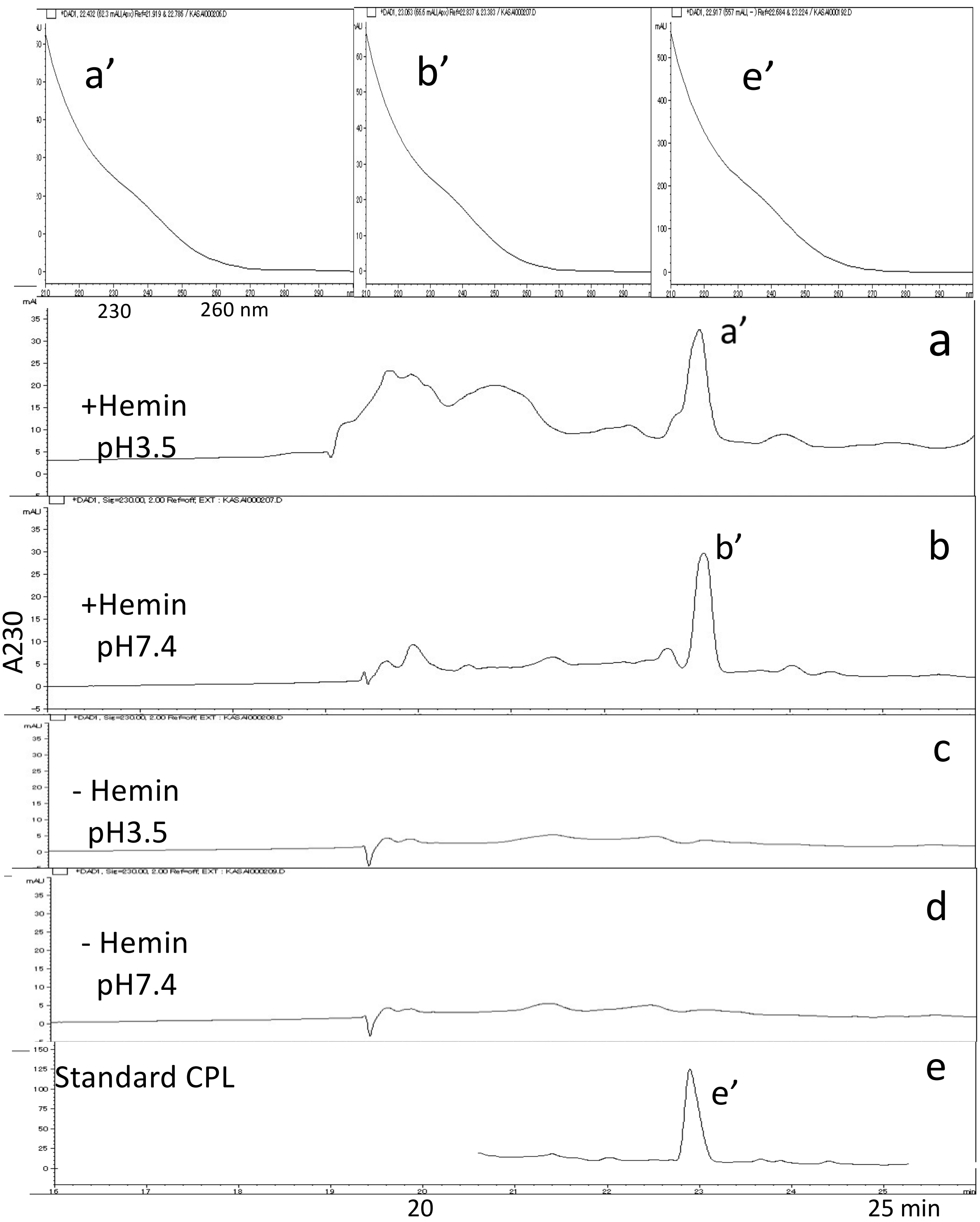

Suppl-4, Formation of CPL from low concentrations of HDE (purified)

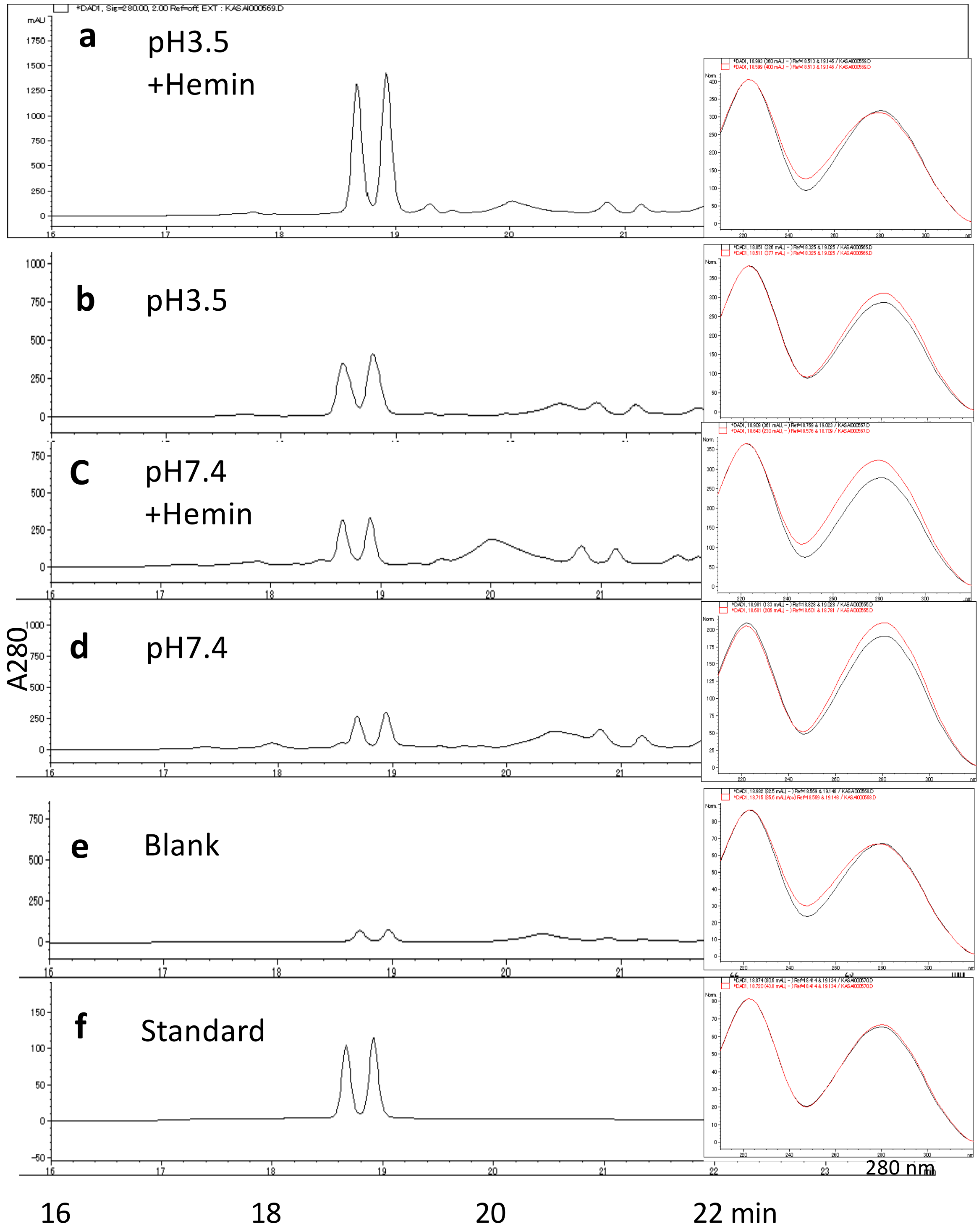

Suppl-5, Formation of BDA-dC adducts from high concentrations of HDE

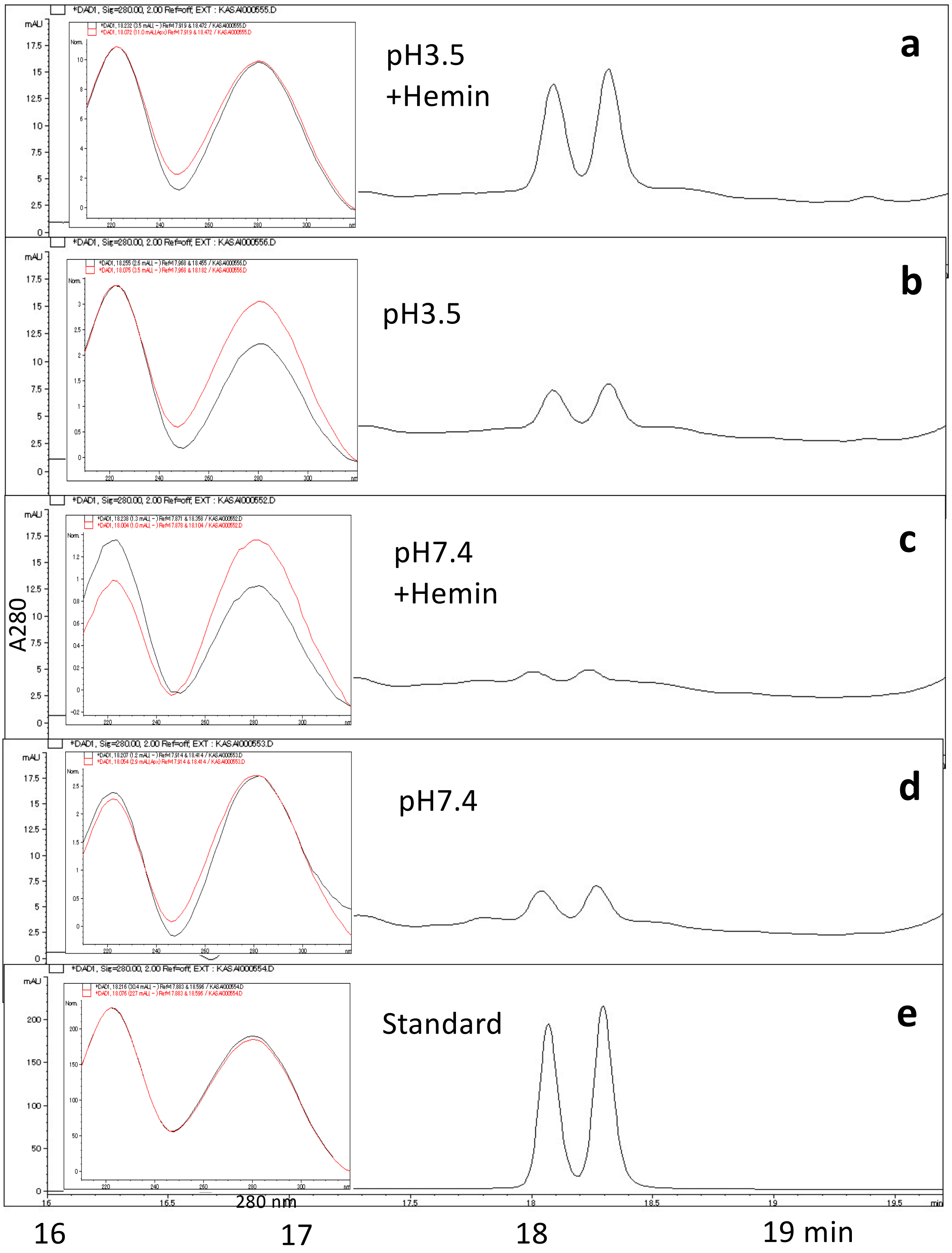

Suppl-6, Formation of BDA-dC adducts from low concentrations of HDE
